# Tree Composition, Niche Characteristics, and Mammal Habitat Use Across Different Types of Forests in Wanglang Nature Reserve

**DOI:** 10.3390/ani16050837

**Published:** 2026-03-07

**Authors:** Chenhui Qu, Chenggong Song, Dongwei Kang, Yanhong Liu

**Affiliations:** School of Ecology and Nature Conservation, Beijing Forestry University, Beijing 100083, Chinasongsuccess9905@bjfu.edu.cn (C.S.);

**Keywords:** forest conservation, species composition, niche characteristic, interspecific correlation, habitat use

## Abstract

Effectiveness of forest restoration efforts depends on the methods employed. Here, we compared tree species composition, niche characteristics, and mammal habitat use in primary, secondary, and artificial forests in Wanglang Nature Reserve. Results showed that primary forests were mainly indicated by *Abies fargesii* var. *faxoniana* (Af), *Picea purpurea* (Pp), and *Juniperus saltuaria* (Js); secondary forests were mainly indicated by Af and *Betula albosinensis* (Ba); and artificial forests were mainly indicated by *Picea asperata* (Pa) and *Acer caesium* (Ac). Af had the broadest niche breadth in natural forests, and Pa had the broadest niche breadth in artificial forests. Low niche overlap among common species was observed in natural forests, whereas high niche overlap between Pa and Ba occurred in artificial forests. Interspecific correlations showed that Af was negatively correlated with Pp in primary forests and *Populus szechuanica* (Ps) in secondary forests. In artificial forests, Af and Ac were positively correlated. Furthermore, no traces of the three National Class I protected species were found in artificial forests, while traces of two representative mammals were associated with Af. These findings highlight the differences among the three types of forests.

## Simple Summary

We elucidated species composition, niche characteristics, and mammal habitat use in primary, secondary, and artificial forests in Wanglang Nature Reserve. These three types of forests were distinguished by different indicator tree species. Natural forests were characterized by low niche overlap, and different interspecific interactions were observed in these three types of forests. No traces of giant panda, Sichuan takin, and golden monkey were found in artificial forests. Accordingly, the protection of natural forests and the improvement of artificial forests should be considered in the conservation of forest habitats of mammals.

## 1. Introduction

Forests harbor most of Earth’s terrestrial biodiversity, providing habitat for approximately 68% of mammal species [1]. However, forest degradation continues at an alarming rate [2], accelerating biodiversity loss [3,4]. Growing awareness of forest conservation has spurred large-scale initiatives, such as China’s Natural Forest Protection Project [5], the Three-North Shelter Forest Project [6], and the expansion of protected areas worldwide [7,8]. Nevertheless, the loss of primary forests is irreversible [9], and the long-term success of restoration ultimately depends on how effectively secondary and artificial forests can recover both the structure and functions of these original ecosystems.

Secondary and artificial forests often inherit certain features of primary forests [10,11,12], but the extent and effectiveness of their recovery vary considerably [13]. Numerous studies have compared primary, secondary, and plantation forests using plant diversity metrics [14,15,16], animal assemblages [17,18,19], and other ecosystem functions [20,21,22]. Primary forests consistently show the highest performance in terms of biodiversity, stability, and ecological function, and naturally regenerated forests generally outperform plantations [23,24], although these attributes tend to improve over time for both secondary and artificial forests [25].

Most previous studies have focused on estimating biodiversity indices [26,27,28], as well as interaction metrics such as niche overlap and interspecific correlations for different forest types [29,30,31], yet plant–animal associations across forest types have received comparatively less attention [32]. These studies have provided foundational information for understanding differences among forest types and the use of forests by animals. However, many fundamental scientific questions remain unresolved. For example, mammals, as seed dispersers and ecosystem engineers [19], respond to tree characteristics such as species, size, and proximity [33], yet their interactions across forest types are not well understood. Forests are often treated as a contextual background [34,35,36], even though tree species composition can shape resource availability and conservation outcomes [37]. As a result, the role of tree species in distinguishing forest types and supporting animal conservation remains unclear, limiting our ability to understand tree–wildlife relationships and to design effective restoration measures.

Wanglang Nature Reserve, which is well known for its giant panda habitat, contains primary, secondary, and artificial forests, which makes it an excellent natural setting for studies of plant–animal associations across forest types. Although previous studies in Wanglang have examined species diversity, the availability of giant panda habitat, and plant community composition [38,39,40], comprehensive evaluations of interspecific interactions or of the preferences of mammals for different forest types are lacking. Moreover, few studies have examined the relationships between tree species and the habitat use patterns of mammals.

Here, we conducted field surveys across different forest types in Wanglang Nature Reserve to determine differences in the composition of tree species among forest types and the tree-specific habitat use of mammals in different forest types. Our objectives were to characterize differences in tree species composition and indicator strength across forest types; describe and compare species’ niche characteristics and interspecific relationships; and evaluate the relationships of forest types and tree species with key mammalian habitat use. Ultimately, our findings shed light on the interactions of mammals with both forest types and tree species and will help enhance the effectiveness of forest conservation strategies.

## 2. Materials and Methods

### 2.1. Study Area

Wanglang Nature Reserve, one of China’s earliest giant panda reserves [41] and currently a core component of Giant Panda National Park, is located in Pingwu County, Sichuan Province, China. The reserve covers 323 km^2^ and ranges in elevation from 2300 m to 4980 m. It retains largely intact original vegetation, with common tree species including *Abies fargesii* var. *faxoniana*, *Picea purpurea*, *Juniperus saltuaria*, *Betula* spp., *Populus* spp., and *Acer* spp., among which *A. fargesii* var. *faxoniana* is the most dominant [42]. Wanglang also supports abundant wildlife, including National Class I protected species, such as the giant panda, Sichuan takin, and golden monkey [43,44].

Before Wanglang was established as a nature reserve in 1965, large areas of forest within its current boundaries had been logged, primarily in the 1950s, especially near rivers and valleys. Plantations were established in some logged areas, primarily using *Picea asperata*. However, silvicultural tending of young stands has been minimal for several decades, particularly for planted tree stands [45].

### 2.2. Field Survey

To investigate tree communities of different forest types, a total of 78 plots (20 m × 20 m) were established in Wanglang Nature Reserve between August and September 2024, including 34 in primary forests, 32 in secondary forests, and 12 in artificial forests (Table S1). Forest type was determined based on published literature [38,40,44,45], guidance from reserve staff, and our field experience. In each plot, trees with a diameter at breast height (DBH) ≥ 5 cm were measured and recorded, including species identity, DBH, height, and crown width. Traces of mammals were recorded, including animal name, trace count, and details of the nearest tree (i.e., tree species, distance, and size).

### 2.3. Data Analysis

#### 2.3.1. Species Composition Analysis

To analyze the species composition in the three types of forests, we generated a tree species list, determined tree richness, and identified exclusive and shared tree species. The similarity in tree species composition between forest types was calculated using the Sørensen index [46]:

SI = 2c/(a + b),(1)

where SI represents Sørensen similarity index, a and b represent the species richness in each community, and c represents the number of shared species in both communities.

To compare the species composition in detail, common species were determined in each forest. The common species were defined as tree species with a relative basal area (RBA) ≥ 5%. The RBA refers to the percentage of the basal area of a tree species to the total basal area of all trees [47]. To verify the representativeness of the common species, their cumulative RBA percentages were calculated in their respective forest types.

To elucidate differences in species composition and the discriminant power of common species across the three types of forests, non-metric multidimensional scaling (NMDS) and indicator species analysis were performed, respectively. For both methods, common species in one forest type were retained across all forest types, even if they were not classified as common species in the others. The NMDS ordination was based on the Bray–Curtis dissimilarity of ranked distances, and the quality of the output was assessed by the stress value, where stress < 0.10 denotes a good fit [48,49]. In addition, 80% confidence ellipses were used to illustrate the core distribution trends of samples from each forest type in the NMDS. The overlap and separation indicate greater similarity and differences in common, respectively. Indicator species analysis was performed following the method of Dufrêne and Legendre [50]; species with indicator values (IndVal) > 0.25 and *p* < 0.05 were considered significant discriminators of forest types. For each common species, we selected the highest IndVal across all forest types as its representative indicator value. Significant indicators were displayed on the NMDS as arrows.

#### 2.3.2. Niche and Interspecific Relationship Analysis

To characterize the resource use of different species, the niche breadths of all common species in each forest were measured using Levins’ index [51]. Due to differences in sample size among forest types, a standardized Levins’ index was used:

(2) $$B_{L}=(\frac{1}{\sum_{j=1}^{r}P_{ij}^{2}}-1)/(r-1),$$

where B_L_ represents standardized niche breadth, P_ij_ represents the ratio of the number of species i utilizing the jth resource site to the total number of species i utilizing all resource sites, and r represents the number of sample plots.

To characterize the resource use relationships among species, the niche overlap between any two common species in each forest was calculated using the Pianka’s index [52]:

(3) $$O_{ik}=\frac{\sum_{j=1}^{r}P_{ij}P_{kj}}{\sqrt{\sum_{j=1}^{r}P_{ij}^{2}\sum_{j=1}^{r}P_{kj}^{2}}},$$

where O_ik_ represents the niche overlap between species i and k; P_ij_ and P_kj_ represent the RBA of tree species i and k in plot j, and r represents the number of sample plots. O_ik_ was classified into two levels: ≤0.50 for low overlap, and >0.50 for high overlap.

To quantify interspecific correlations, the correlation coefficients between any two common species in each forest were calculated using Spearman’s correlation coefficient (r_S_) [53]:

(4) $$r_{S}(i,k)=1-\frac{6\sum_{j=1}^{N}{\left(x_{ij}-x_{kj}\right)}^{2}}{N^{3}-N},$$

where N represents the total number of plots and x_ij_ (x_kj_) represents the RBA of the species in plot i(k). The |r_S_| was classified into two levels: |r_S_| < 0.50 for weak correlations and |r_S_| > 0.50 for strong correlations. The threshold for statistical significance was *p* < 0.05. Our principal focus was on significant and strong correlations between species pairs.

#### 2.3.3. Mammal Habitat Use Analysis

We analyzed the occurrence of mammal traces to evaluate habitat use among forest types. Specifically, we calculated the proportion of plots with mammal traces (i.e., the proportion of plots with traces to the total number of plots). Protected animals were identified according to the List of National Key Protected Wild Animals (2021) [54].

To describe the habitat use of representative mammals in relation to tree species, we analyzed the nearest trees to their traces. Representative mammals were defined as those occurring in ≥20% of all plots. For each trace, the nearest tree was identified, and its species name and four variables, distance, DBH, height, and crown width, were recorded. The proportion of nearest trees was calculated as the percentage of individuals of a given tree species relative to the total number of nearest-tree records. Mean values of the recorded variables were calculated within each forest type, and differences among forest types were tested using one-way analysis of variance [55]; the threshold for statistical significance was *p* < 0.05.

## 3. Results

### 3.1. Species Composition

A total of 45 tree species were recorded, with 25, 37, and 29 species recorded in the primary, secondary, and artificial forest plots, respectively. A total of 15 species were shared across all three forest types (Table S2). The similarity index between secondary and artificial forests was the highest (SI = 0.89), followed by the index between primary and secondary forests (SI = 0.71) and that between primary and artificial forests (SI = 0.56).

Based on the standard of RBA ≥ 5%, we identified three common species of primary forest (Af, Pp, and Js), with a cumulative RBA value of 94.9%; three common species of secondary forest (Af, Ba, and Ps), with a cumulative RBA value of 79.8%; and five common species of artificial forest (Pa, Af, Bp, Ac, and Ba), with a cumulative RBA value of 69.1% (Table 1).

**Figure 1:**
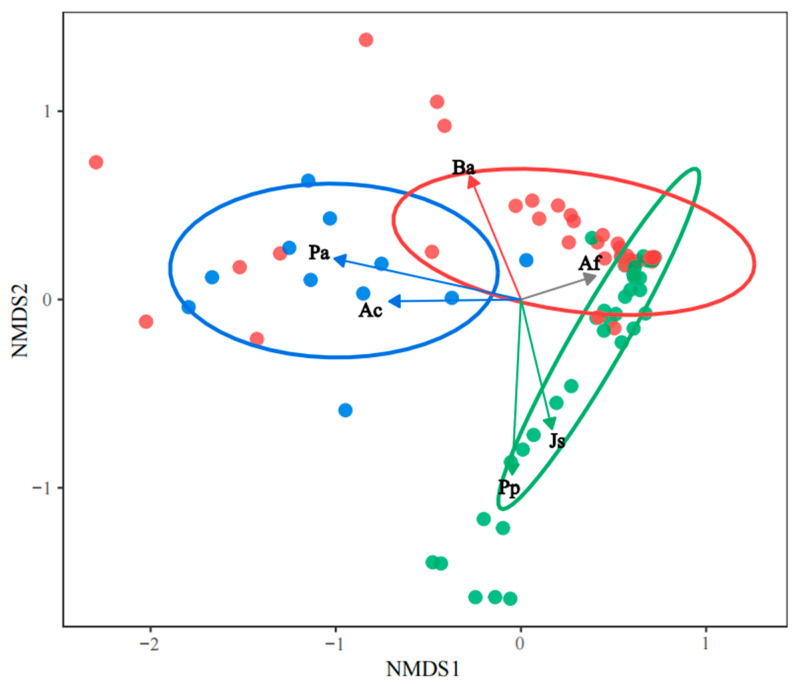
Non-metric multidimensional scaling (NMDS) of common tree species across three forest types. stress = 0.08 < 0.10. Colors denote forest categories: green, primary forests; red, secondary forests; blue, artificial forests; and gray, shared by primary and secondary forests. Af: *Abies fargesii* var. *faxoniana*; Pp: *Picea purpurea*; Js: *Juniperus saltuaria*; Ba: *Betula albosinensis*; Pa: *Picea asperata*; Ac: *Acer caesium*.

According to the NMDS ordination (stress = 0.08), the artificial forest was clearly separated from the primary forest, whereas the secondary forest partially overlapped with primary and artificial forests (Figure 1).

Six of the eight common species were identified as significant indicator species among the three forest types. Af (IndVal = 0.61, *p* < 0.01) served as a shared indicator species of both primary and secondary forests. In the primary forests, Pp (IndVal = 0.55, *p* < 0.01) and Js (IndVal = 0.43, *p* < 0.01) were additional indicator species, whereas Ba (IndVal = 0.36, *p* < 0.05) was an indicator of secondary forests. The artificial forests were indicated by Pa (IndVal = 0.76, *p* < 0.01) and Ac (IndVal = 0.33, *p* < 0.05) (Table 2).

### 3.2. Niche Characteristics and Interspecific Correlations

**Figure 2:**
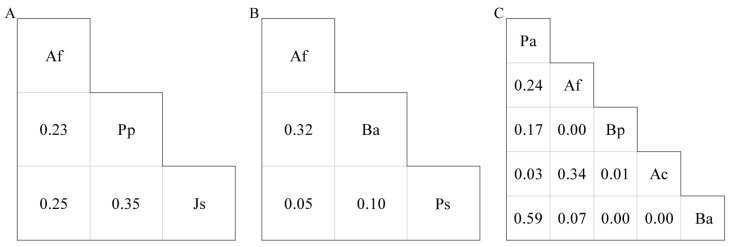
Pianka niche overlap index. (**A**): primary forests; (**B**): secondary forests; (**C**): artificial forests. Af: *Abies fargesii* var. *faxoniana*; Pp: *Picea purpurea*; Js: *Juniperus saltuaria*; Ba: *Betula albosinensis*; Ps: *Populus szechuanica*; Pa: *Picea asperata*; Bp: *Betula platyphylla*; Ac: *Acer caesium*.

In primary forests, Af had the broadest niche breadth, followed by Pp and Js (Table 1). The niche overlap values between these species pairs were <0.50 (Figure 2A). In secondary forests, Af had the broadest niche breadth, followed by Ba and Ps (Table 1). The niche overlap values between these species pairs were <0.50 (Figure 2B). In artificial forests, Pa had the broadest niche breadth (Table 1). The niche overlap value of Pa-Ba was >0.50, while those of other species pairs were <0.50 (Figure 2C).

**Figure 3:**
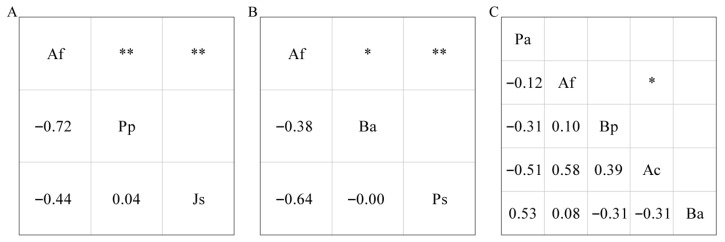
Spearman correlation coefficients and significance levels. (**A**): primary forests; (**B**): secondary forests; (**C**): artificial forests; * *p* < 0.05, ** *p* < 0.01. Af: *Abies fargesii* var. *faxoniana*; Pp: *Picea purpurea*; Js: *Juniperus saltuaria*; Ba: *Betula albosinensis*; Ps: *Populus szechuanica*; Pa: *Picea asperata*; Bp: *Betula platyphylla*; Ac: *Acer caesium*.

Af was strongly negatively correlated with Pp (|r_S_| > 0.50, *p* < 0.01) in primary forests (Figure 3A) and Ps (|r_S_| > 0.50, *p* < 0.01) in secondary forests (Figure 3B). In artificial forests, Af and Ac were strongly positively correlated (|r_S_| > 0.50, *p* < 0.05), and no significant correlations were observed for other species pairs (*p* > 0.05) (Figure 3C).

### 3.3. Habitat Use of Mammals Related to Forests and Trees

A total of 90 traces from 12 mammal species were recorded in 37 plots, with 6, 10, and 6 mammal species in primary, secondary, and artificial forests, respectively. The proportion of plots with traces was highest in primary forest (52.9%), followed by secondary (46.9%) and artificial forests (38.5%).

A total of three national first-class protected animals’ traces were recorded, traces of giant panda and golden monkey were recorded in secondary forest plots, and traces of Sichuan takin were found in both primary and secondary forest plots. None of their traces were found in artificial forests. Two mammals, *Naemorhedus griseus* (Ng) and *Elaphodus cephalophus* (Ec), in the order Cetartiodactyla, were determined as representative mammals that their traces were detected in ≥20.0% of all plots (33.3% and 22.8%, respectively; Table S3).

The most common nearest tree species was Af for both Ng and Ec. The most common nearest tree species to Ng in the primary and secondary forest types was Af (50.0% vs. 75.0%), while that in the artificial forests was *Rhamnus sargentiana* (50.0%) (Table S4).

The most common nearest tree species to Ec in the primary and secondary forest types was Af (57.1% vs. 70.0%); in artificial forests, the most common nearest tree species to Ec was Pa (40.0%) (Table S5).

For both Ng and Ec, no significant differences were observed in the four variables of the nearest trees across forest types (*p* > 0.05; Table 3).

## 4. Discussion

### 4.1. Variation in Species Composition Across Forest Types

Species composition is an important attribute of plant communities [56]. Trees are a fundamental component of forest ecosystems, as they play critical roles in shaping community structure and providing essential habitats [57]. We characterized the performance of three types of communities in Wanglang Nature Reserve, primary, secondary, and artificial forests, based on analysis of the richness, similarity, composition, and importance of tree species.

Species richness is an important indicator of species composition [23]. The species composition of forest types typically varies. Many studies have demonstrated variation in the tree species richness of forests with different origins [15,16,58,59]. Patterns of tree richness observed in the three types of forests in our study were consistent with the results of several similar studies [16,38,60].

Species similarity among forest types also provides insights into species composition [25]. The number of shared species among the three forest types was 15, which accounted for one-third of the recorded tree species in our study and contributed to the high SI values among forest types. For example, the similarity between primary and secondary forests and between secondary and artificial forests was >0.70, while that between primary and artificial forests was <0.60. These results were consistent with the findings of previous studies in this reserve [38,60]. Secondary forests are formed by natural recovery after logging and are thus more similar to primary forests; by contrast, artificial forests are derived from artificial planting and thus differ more markedly from primary forests. Furthermore, the similarity between secondary and primary forests could reflect early floristic convergence, and the low similarity of the planted forest may be due to both the origin of the plantation and the homogeneous age of the stand. Therefore, management, floristic, and stand status may have contributed to the observed variation in species similarity [23,60,61].

Common species can have a major effect on species composition [62,63]. In Wanglang, Af, Pp, and Js are the dominant tree species in the three typical climax coniferous forest communities [44,45,64], and they were the common species in primary forests identified in our study. After decades of natural recovery after logging, the secondary forest communities have generally reached a mid-successional stage with various local tree species, such as Af, Ba, and Ps [65]. However, Pa was the main afforestation tree species [39]. Pa is an important common species in artificial forests due to its large-scale and high-density planting. Furthermore, Pa, along with some native tree species that were previously present or subsequently invaded, were the common species in artificial forests, which stems in part from the shift in forest management from timber production to ecological protection [8,66]. This helps understand the variation in the composition of common species in the three forest types.

Based on the NMDS ordination and indicator analysis, the three forest types were distinguished by six species. Pa and Ac were the two species distinguishing artificial forests from natural forests (i.e., primary and secondary forests). Af was the common indicator species associated with natural forests, whereas Pp and Js were the indicator species of primary forests, and Ba was the indicator species of secondary forests. Our findings provide an important foundation for distinguishing different types of forests.

### 4.2. Relationships Between Common Species Across Different Forest Types

A forest community comprises multiple interacting populations of species [67]. Studies of the survival of these species and the relationships among these species can reveal distinct patterns of community structure and assembly mechanisms [68,69]. We characterized the attributes of three types of forest communities in Wanglang Nature Reserve based on niche characteristics and interspecific correlations.

Niche characteristics are central to understanding community structure [70]. Niche breadth measures the range of resource use by species, while niche overlap measures the similarity and potential competition in resource use among species [71]. Af had the broadest niche breadth in both primary and secondary forests, which is consistent with its status as a foundation species with high resource use in the reserve [44]. In contrast to the dominant native species Af, Pa had the broadest niche breadth in artificial forests, which reflects their history of large-scale plantation establishment. Furthermore, niche overlap among species pairs was low in natural forests but high between Pa and Ba in artificial forests. In artificial forests, the pioneer species Ba showed high niche overlap with the planted species Pa.

Species correlations reflect interspecific relationships to some extent [72]. In natural forests, Af was significantly negatively correlated with Pp and Ps in primary and secondary forests, respectively. Similar correlations between dominant species in natural forests have been reported in previous studies [73,74,75]. However, the mechanisms underlying these relationships may be different. In primary forests, Af and Pp, two key evergreen conifers, each comprised separate climax communities in Wanglang [76,77,78]. The mean RBA of Af or Pp exceeded 75% among plots dominated by either species, and only three plots contained both species at comparable RBAs. In secondary forests, which represent mid-successional stages after logging, not all species have fully recovered across all sites [12]. In five plots dominated by the pioneer species Ps, Af failed to re-establish; this is consistent with the significantly negative correlation observed between Ps and Af. In artificial forests, except Af was significantly positively correlated with Ac, most interspecific correlations were not significant; this phenomenon may related to anthropogenic activity [26,73,79].

Our study revealed the internal characteristics of forest communities based on niche characteristics and interspecific correlations. These findings provide a more comprehensive understanding of community structure compared with species composition alone. However, species composition and community structure are affected by environmental factors (i.e., meteorology, terrain, and soil) [80]. Although the influence of environmental conditions is not the focus of this study, the observed patterns may have also been modulated by environmental conditions. Thus, differences between forest types reflect a combination of stand origin, historical management, and environmental gradients not explicitly assessed.

### 4.3. Forest Type and Tree Species Related to Mammal Habitat Use

Forests provide diverse habitats for wildlife [81]. Habitat use of mammals reflects habitat quality and drive patterns of habitat selection [82]. Animal traces serve as indirect indicators of species presence and are widely used to assess habitat use and distribution patterns [83]. In this study, the proportion of plots with mammal traces was higher in primary and secondary forests but lower in artificial forests, and no traces of giant panda, Sichuan takin, and golden monkey, were recorded in artificial forests. These results indicate that although artificial forests can be used, mammals make more use of natural forests. This, to some extent, reflects the low habitat functionality of artificial forests for some local mammal species [60,84,85,86,87,88].

The plant–animal relationship can reflect the habitat requirements of species [89]. Large trees are crucial for mammalian habitat use [90,91,92]. However, neither Ng nor Ec exhibited significant differences in tree size and distance across forest types in this study; instead, they both were associated with Af, especially in natural forests. These patterns suggest that natural forests dominated by Af supported a broader range of habitat conditions, and the importance of Af in constituting the habitats of these two animals.

### 4.4. Suggestions, Limitations, and Future Research Directions

We identified differences in species composition and community structure among the three forest types. Priority should be given to protecting primary forests, while continuing the recovery of secondary forests and promoting the restoration of artificial forests to enhance the habitat function of forest communities in Wanglang.

Our study has some limitations that require consideration. For example, the state of forests with different origins (i.e., primary, secondary, and artificial forests) is not static but dynamic, the observed differences thus only represent a specific temporal state. Furthermore, habitat use data inferred from mammal traces in different forest plots reflects relative use from a specific perspective rather than absolute preference, and it may also vary with time (i.e., monthly, seasonal, and yearly) and under different environmental conditions, being conditional. Therefore, additional studies based on long-term monitoring data and various investigation methods are needed to further enhance the reliability of the conclusions.

## 5. Conclusions

We analyzed species composition, niche characteristics, and mammal habitat use in primary, secondary, and artificial forests in Wanglang Nature Reserve. Three types of forests were distinguished by different indicator tree species. Natural forests were characterized by low niche overlap, and different interspecific interactions were observed in these three types of forests. Furthermore, no traces of the three National Class I protected species were found in artificial forests, and traces of two representative mammals were associated with Af. These findings suggest that several actions should be taken for forest conservation, including the protection of primary forests, the continued recovery of secondary forests, and the transformation of artificial forests.

## Figures and Tables

**Table 1:** Common tree species composition and niche breadth in three types of forests.

Forest Type	Species (Abbreviation)	RBA ^1^ Value (%)	B_L_ ^2^ Value
Primary forest	*Abies fargesii* var. *faxoniana* (Af)	56.24	0.75
	*Picea purpurea* (Pp)	29.68	0.40
	*Juniperus saltuaria* (Js)	9.01	0.23
Secondary forest	*Abies fargesii* var. *faxoniana* (Af)	63.06	0.72
	*Betula albosinensis* (Ba)	9.34	0.29
	*Populus szechuanica* (Ps)	7.36	0.14
Artificial forest	*Picea asperata* (Pa)	40.04	0.53
	*Abies fargesii* var. *faxoniana* (Af)	9.01	0.12
	*Betula platyphylla* (Bp)	8.69	0.00
	*Acer caesium* (Ac)	6.33	0.08
	*Betula albosinensis* (Ba)	5.05	0.12

^1^ RBA: relative basal area; ^2^ B_L_: Levins’ index.

**Table 2:** Indicator species across three forest types.

No.	Species	Forest Type			Indicator Value	*p* Value
		Primary Forest	Secondary Forest	Artificial Forest		
1	*Abies fargesii* var. *faxoniana* (Af)	√	√		0.61	0.00
2	*Picea purpurea* (Pp)	√			0.55	0.00
3	*Juniperus saltuaria* (Js)	√			0.43	0.00
4	*Betula albosinensis* (Ba)		√		0.36	0.04
5	*Populus szechuanica* (Ps)		√	√	0.24	0.13
6	*Picea asperata* (Pa)			√	0.76	0.00
7	*Betula platyphylla* (Bp)			√	0.21	0.17
8	*Acer caesium* (Ac)			√	0.33	0.03

√: Indicator species for the forest type.

**Table 3:** Nearest tree characteristics related to *Naemorhedus griseus* and *Elaphodus cephalophus* across three forest types.

Species	Variable	Mean (Standard Deviation)			F Value	*p* Value
		Primary Forest	Secondary Forest	Artificial Forest		
*Naemorhedus griseus*	Distance (m)	1.74 (0.95)	1.43 (0.60)	1.45 (0.52)	0.56	0.58
	DBH (cm)	24.25 (22.58)	20.11 (16.2)	23.49 (3.79)	0.16	0.85
	Height (m)	10.39 (6.67)	9.58 (3.65)	8.38 (2.43)	0.25	0.78
	Crown breadth (m^2^)	10.71 (7.65)	5.66 (4.52)	13.76 (10.20)	2.76	0.08
*Elaphodus cephalophus*	Distance (m)	1.89 (1.06)	1.72 (0.87)	1.86 (0.69)	0.32	0.73
	DBH (cm)	46.88 (27.61)	29.57 (15.71)	29.55 (24.27)	1.49	0.25
	Height (m)	14.14 (5.37)	11.45 (4.07)	7.90 (6.27)	2.26	0.13
	Crown breadth (m^2^)	19.48 (14.46)	6.41 (3.30)	14.34 (16.91)	2.77	0.09

## Data Availability

The original contributions presented in this study are included in the article/Supplementary Material. Further inquiries can be directed to the corresponding author.

## Supplementary Materials

The following supporting information can be downloaded at: https://www.mdpi.com/article/10.3390/ani16050837/s1, Table S1. Sample plot basic information; Table S2. Exclusive or shared species across three forest types; Table S3. Proportion of plots with mammal traces; Table S4. The nearest tree species to *Naemorhedus griseus* across three forest types; Table S5. The nearest tree species to *Elaphodus cephalophus* across three forest types.
