# Supplementary material for: Tree Composition, Niche Characteristics, and Mammal Habitat Use Across Different Types of Forests in Wanglang Nature Reserve
Source: Animals (Basel). 2026 Mar 7;16(5):837. doi: 10.3390/ani16050837 (PMC12985036; doi:10.3390/ani16050837)
Supplement: Supplementary file 1 [file animals-16-00837-s001.zip › animals-4139906-supplementary.pdf]

Supplementary materials

Table S1. Sample plot basic information.

Table S2. Exclusive or shared species across three forest types.

Table S3. Proportion of plots with mammal traces.

Table S4. The nearest tree species to *Naemorhedus griseus* across three forest types.

Table S5. The nearest tree species to *Elaphodus cephalophus* across three forest types.

Table S1. Sample plot basic information.

Forest Type	Plot number	Longitude (°)	Latitude (°)	Elevation (m)
Primary forest	1	103.995787	32.962631	2983
	2	103.997701	32.967243	3015
	3	104.001503	32.971769	3018
	4	104.004299	32.978686	2957
	5	104.005496	32.984332	2871
	6	104.007122	32.990021	2853
	7	104.009067	32.994523	2880
	8	104.012961	32.997617	2883
	9	104.017398	33.001391	2846
	10	104.023319	33.004035	2838
	11	104.025113	33.017550	2974
	12	104.026122	33.007068	2897
	13	104.026128	32.874258	3166
	14	104.026253	33.013987	2886
	15	104.032973	32.876822	3108
	16	104.035742	32.930915	3071
	17	104.035861	33.002882	2859
	18	104.040067	32.880413	3062
	19	104.040577	33.000641	2784
	20	104.040905	32.933005	3077
	21	104.044960	32.882545	3066
	22	104.045933	32.936685	2979
	23	104.048022	32.893500	3002
	24	104.048467	32.880365	3064
	25	104.048522	32.887957	3006
	26	104.051288	32.898612	2966
	27	104.051705	32.938050	2920
	28	104.053325	32.910388	2967
	29	104.053798	32.903220	2970
	30	104.055617	32.906870	2959
	31	104.058180	32.913233	2923
	32	104.058665	32.939195	2825
	33	104.061000	32.918408	2903
	34	104.063360	32.923230	2730
Secondary forest	35	104.030542	33.003945	2836
	36	104.043975	32.996303	2780
	37	104.049025	32.999420	2859
	38	104.049190	32.990470	2710
	39	104.052102	32.874968	3116
	40	104.055261	32.987437	2681
	41	104.060692	32.987886	2685
	42	104.060932	32.933837	2811
	43	104.062027	32.929563	2814
	44	104.065432	32.945925	2729
	45	104.065527	32.942645	2731
	46	104.067352	32.949778	2735

Forest Type	Plot number	Longitude (°)	Latitude (°)	Elevation (m)
	47	104.068252	32.953922	2814
	48	104.068385	32.959833	2655
	49	104.068838	32.964200	2655
	50	104.071524	32.983194	2616
	51	104.072163	32.968445	2630
	52	104.073530	32.973098	2681
	53	104.083555	32.978382	2563
	54	104.088647	32.976480	2612
	55	104.089761	32.970951	2606
	56	104.100897	32.974969	2834
	57	104.102312	32.977454	2927
	58	104.112086	32.965755	2536
	59	104.116688	32.964843	2537
	60	104.125479	32.910606	2546
	61	104.127939	32.951514	2487
	62	104.128357	32.915062	2602
	63	104.133094	32.920953	2518
	64	104.137828	32.927863	2484
	65	104.138368	32.936192	2476
	66	104.145335	32.926670	2457
Artificial forest	67	104.067100	32.986092	2658
	68	104.076467	32.978850	2595
	69	104.080053	32.979240	2577
	70	104.096149	32.970483	2515
	71	104.102084	32.971926	2648
	72	104.107164	32.969325	2563
	73	104.113578	32.967650	2526
	74	104.119736	32.960257	2501
	75	104.122856	32.954704	2541
	76	104.127936	32.951514	2497
	77	104.136984	32.943848	2487
	78	104.155725	32.912705	2407

Table S2. Exclusive or shared species across three forest types.

Species type	Forest type	Number (Proportion)
Shared species	All forest types	15 (33.3%)
	Primary and secondary forest	7 (15.6%)
	Primary and artificial forest	0 (0%)
	Secondary and artificial forest	9 (20.0%)
Exclusive species	Primary forest	3 (6.7%)
	Secondary forest	6 (13.3%)
	Artificial forest	5 (11.1%)

Table S3. Proportion of plots with mammal traces.

Classification (Order)	Species	Proportion of plot
Cetartiodactyla	*Naemorhedus griseus*	33.3%
	Elaphodus cephalophus	22.8%
	Budorcas tibetanus	7.0%
	Capricornis milneedwardsii	7.0%
	Muntiacus reevesi	8.8%
	Moschus berezovskii	3.5%
	Pseudois nayaur	1.8%
	Sus scrofa	1.8%
Carnivora	*Ailuropoda melanoleuca*	5.3%
	Arctonyx collaris	3.5%
	Martes flavigula	1.8%
Primates	*Rhinopithecus roxellana*	3.5%

Table S4. The nearest tree species to *Naemorhedus griseus* across three forest types.

Species	Proportion			
	Primary forest	Secondary forest	Artificial forest	Total
Abies fargesii var. *faxoniana*	50.0%	75.0%		53.3%
Juniperus saltuaria	14.3%			6.7%
Acer caudatum	14.3%			6.7%
Picea purpurea	7.1%			3.3%
Sorbus koehneana	7.1%			3.3%
Rhododendron watsonii	7.1%			3.3%
Betula utilis		8.3%		3.3%
Philadelphus incanus		8.3%		3.3%
Salix wallichiana		8.3%		3.3%
Rhamnus sargentiana			50.0%	6.7%
Pinus armandi			25.0%	3.3%
Euonymus frigidus			25.0%	3.3%

Table S5. The nearest tree species to *Elaphodus cephalophus* across three forest types.

Species	Proportion			
	**Primary forest**	**Secondary forest**	**Artificial forest**	**Total**
Abies fargesii var. *faxoniana*	57.1%	70.0%	20.0%	54.6%
Picea purpurea	28.6%			9.1%
Salix rehderiana var. *dolia*	14.3%			4.6%
Betula albosinensis		10.0%		4.6%
Sorbus koehneana		10.0%		4.6%
Prunus obtusata		10.0%		4.6%
Picea asperata			40.0%	9.1%
Tilia amurensis			20.0%	4.6%
Euonymus frigidus			20.0%	4.6%
